# Multiple Health Behavior Programs in School Settings: Strategies to Promote Transfer-of-Learning Through Life Skills Education

**DOI:** 10.3389/fpubh.2021.716399

**Published:** 2021-08-24

**Authors:** Veronica Velasco, Corrado Celata, Kenneth W. Griffin, Estensione LST group

**Affiliations:** ^1^Psychology Department, Università degli Studi di Milano-Bicocca, Milan, Italy; ^2^Health Promotion Division, Specific Prevention Unit, Agenzia di Tutela della Salute (ATS) Milano Città Metropolitana, Milan, Italy; ^3^Department of Global and Community Health, George Mason University, Fairfax, VA, United States; ^4^Health Promoting School–Lombardy, Milan, Italy

**Keywords:** school health promotion, transferability, evidence-based program, multiple health behavior change, life skill

## Abstract

Typically, schools implement health promotion programs that focus on a single behavioral domain. Multiple related health topics may be addressed using separate interventions, potentially producing overlap in program content. However, integrative approaches in health promotion have the potential to produce interventions capable of improving multiple health behaviors. In particular, more research is needed to identify the conditions and the factors that can promote the transfer of learning to broaden the target outcomes of health promotion programs. The present study aims to identify the characteristics of an evidence-based life skills education program that can facilitate the transfer of learning to different health behaviors not initially targeted by the program, and the strategies for achieving successful transfer. A two round Delphi method was used with a diverse group of 21 experts in health promotion, life skills education, and methods of pedagogy for early adolescent students. Questionnaires with open and closed-ended questions were administered on-line. Content qualitative analysis was run, integrating codes, subcategories, and categories of the two rounds of the study. Results showed strong consensus among experts about the potential for promoting the transfer of skills from one health domain to another. Many elements were identified as important factors that facilitate the transfer of learning. Strategies for successful transfer were related to teaching methods, educational approaches, and consistency with the broader school curriculum. Findings suggest that the successful transfer of learning to a new health domain requires that educators recognize its importance and explicitly designate it as an educational aim.

## Introduction

Typically, schools implement health promotion programs that focus on a single behavioral domain, and multiple related health topics may be addressed using separate interventions (1). This may occur because educators often view student health in the context of specific behaviors and outcomes; they also tend to hold beliefs about health promotion that are problem-specific rather than broad-based (2, 3). Moreover, when new problem behaviors are observed, there may be public pressure from families and local communities to address the issue promptly. Schools may then choose to implement a new prevention program that addresses the problem, but this may further increase an overlap in content areas across potentially similar interventions. For example, in recent years, new interventions have been developed to prevent youth gambling (4), reduce problem internet use (5), and promote mindful meditation (6), yet when new programs are adopted and implemented there may be little consideration given to whether the new content overlaps with existing school programming. Also, most evidence-based programs focus on specific health domains and their evaluations typically focus only on those specific behavioral outcomes. Crossover effects, in which researchers examine the impact of an intervention on similar behaviors with similar etiologies, are rarely tested. When overlapping interventions are implemented simultaneously, this may contribute to logistical problems and the inefficient use of limited resources. Every intervention requires funding, time, and capacity-building, and school staff can become overwhelmed with the numerous requests they receive to implement social and health-related interventions (7–12).

An important potential strategy to help reduce programmatic overlap may be to assist schools in identifying their specific needs for prevention and guide them in selecting the most appropriate interventions to implement (12). However, if only specific, highly prevalent problem behaviors are considered, this will fail to address the importance of primary and universal prevention and the promotion of healthy behaviors and well-being, which by definition should be implemented prior to any specific need. It is also important to consider that health behaviors are often related to one another, influence each other, and are related to people's lifestyles (13–16). Multiple unhealthy or risky behaviors often co-occur in both adolescents (17, 18) and adults (19). Furthermore, multiple health risk behaviors, including alcohol, drug use, and sexual risk taking, share a common set of risk and protective factors that include sociodemographic, interpersonal, school and family factors (20).

Thus, another strategy to reduce programmatic overlap may be to target multiple behaviors with the same intervention. In 2002, the Society of Behavioral Medicine formed a special interest group to contribute to the development of a science of multiple health behavior change (MHBC) for health promotion and disease management (19). MHBC has since become a developed area of research with theoretical and practical implications. Studies about the potential for long-term effects of broad-based prevention programs on behaviors that were not explicitly targeted as part of an MHBC intervention have been published and reviewed (8, 12, 21–24). A comprehensive and holistic approach, as opposed to one focusing on a single dimension of child health, has been supported for over 25 years by the whole school approach and the Health Promoting Schools (HPS) framework of the World Health Organization (WHO) (25–29).

Although the importance of the MHBC approach is recognized, there is little consensus on how to most effectively combine multiple health promotion programs with potentially overlapping content (8, 19, 30). An integrative approach is a promising framework for developing MHBC interventions (12, 13, 19, 23, 31–34). It aims to integrate intervention content based on links between health behaviors and their determinants (35, 36). Several health behavior theories suggest that distal psychosocial determinants are the same for a variety of different healthy and risky behaviors. Other theories identify proximal determinants which are often domain-specific but are generalizable constructs (e.g., self-efficacy, attitudes, and social influences).

An integrative strategy suitable for school settings is the *transfer of learning* approach, an approach based on extensive educational theory and research (37–43). Transfer of learning “*occurs when learning in one context or with one set of materials impacts on performance in another context or with other materials*” (42 p. 3). Transfer of learning is a core concept in education because it represents the final aim of education, that is, producing students with the capacity to apply acquired knowledge and skills to new situations and contexts (42). For example, students learn to interpret a textbook, then a romance novel or an essay, and in the long term, these literacy and critical thinking skills transfer to related activities and should enable them to read a newspaper critically. This transfer will also improve students' citizen skills and behaviors. Transfer of learning occurs across several dimensions that can be distinguished from one another (37). These include the specific context of the initial instruction, characteristics of the situation where this learning that takes place, the content of what was first learned and how it is transferred to a new context, the performance that is required in the original learning and new applied contexts, and the level of specificity and generality of the content and contexts. The contextual differences include several aspects: learning can be transferred across different knowledge domains or across varying social, physical, or temporal contexts. The change in these contexts can be small (e.g., at school for the same subject, for a different subject, or for a laboratory activity) or large (e.g., the transfer of learning from school to at home, or to applications when interacting with friends outside of school). These classifications show how powerful and widespread the transfer of learning can be.

A similar transfer of learning process is likely to occur as a result of participating in health promotion interventions, particularly when such programs are based on a positive youth development framework. Examining the transfer of learning in health promotion instruction is important yet understudied. Students may learn knowledge, attitudes, and skills in one context or health behavior domain and then be able to apply them in other contexts or domains (12, 44–46). For example, problem-solving or assertiveness skills can be developed in a drug prevention context and then be transferred to scenarios involving reducing sexual risk behaviors. Similarly, awareness about the influence of social norms or the adoption of healthy values may be also transferred from one health behavior domain to another (8, 12, 21, 22, 24). For school-based health promotion, a promising approach for MHBC is life skill education. This broad-based approach focuses on general skills building and enhancing resilience and has proven to be an effective prevention strategy across multiple health risk behaviors (47, 48). Life skills prevention approaches are based on broad theories, such as Problem Behavior Theory (49) or Social Learning Theory (50), that are applied to multiple behavioral domains. Methods and strategies focus on building and developing cognitive, emotional and behavioral skills. Action learning and cooperative learning methodologies are used and have been shown to be effective. The teaching methods are flexible and can be adapted for a variety of contexts or student characteristics. Life skill education efficacy increases when implemented through a whole school approach (51–53). Indeed, most of the programs included in reviews about multiple behavior approaches use a life skills education strategy (8). In practice, the transfer of context is a core concept for many health promotion and life skills education programs. Skills are taught to students prior to situations where they may be called upon to use them. For example, students' refusal skills are taught and reinforced from an early age so that students can later use these skills to refuse cigarette offers as adolescents.

A research area that needs more attention is the definition of the conditions that can promote the transfer of learning. Transfer of learning does not occur without the sustained attention by stakeholders to several necessary preconditions (12, 34, 42, 54, 55). Paulussen et al. identified three preconditions (34): behaviors are associated and have similar determinants, methods to modify these determinants are similar, and students are encouraged to apply what they have learned to different behaviors. Peters (12) underlines two transfer-promoting aspects. First, general principles or procedures relevant for carrying out behaviors must be explicitly addressed (e.g., rules about how to refuse), and students must be prompted to apply these general principles to multiple health behavior domains (e.g., tobacco and sex). Processes of contextualization (learning new skills in one context), decontextualization (deducing a general principle), and recontextualization (examining application in other contexts) should be both knowable and identifiable. Second, these general principles must be meaningful to students and relevant to their personal lives. Perkins and Salomon (42) outline several different conditions in which transfer of learning occurs, the mechanisms that underlie such transfer, and how strategies to promote transfer should be taught. What they refer to as the “low road of transfer” is based on repeated practice in different contexts, where practice activates specific scripts, action schema, and semi-automatic responses, and the transfer happens when stimulus conditions are similar to those in a prior context of learning and are able to trigger the same responses. What they call the “high road of transfer” is promoted by mindful abstraction, metacognition, and deliberate search of connections. The integration of these two approaches should be considered in developing a transfer of learning approach in the health promotion area.

### Purpose

The present study focuses on the transfer of learning approach in health promotion. It aims to identify the conditions and factors that can facilitate the transfer of learning during the implementation of a life skills education program. In particular, the objectives are to identify:

- the characteristics of a life skill education program that can facilitate the transfer of learning to different health behaviors not explicitly targeted by the program,
- the necessary elements that should be reinforced to facilitate the transfer, and
- the strategies for achieving successful transfer.

The Delphi method was used to solicit input on these issues from a diverse group of experts. The Delphi method (56–59) is a consensus development technique used to obtain the most reliable consensus of a group of experts. In health research, Delphi methods have been used to identify core outcomes to measure in randomized controlled trials (RCTs), to identify research priorities in a specific setting, and to develop guidelines and specify program theory (60–64).

## Method

### Design

**Figure 1:**
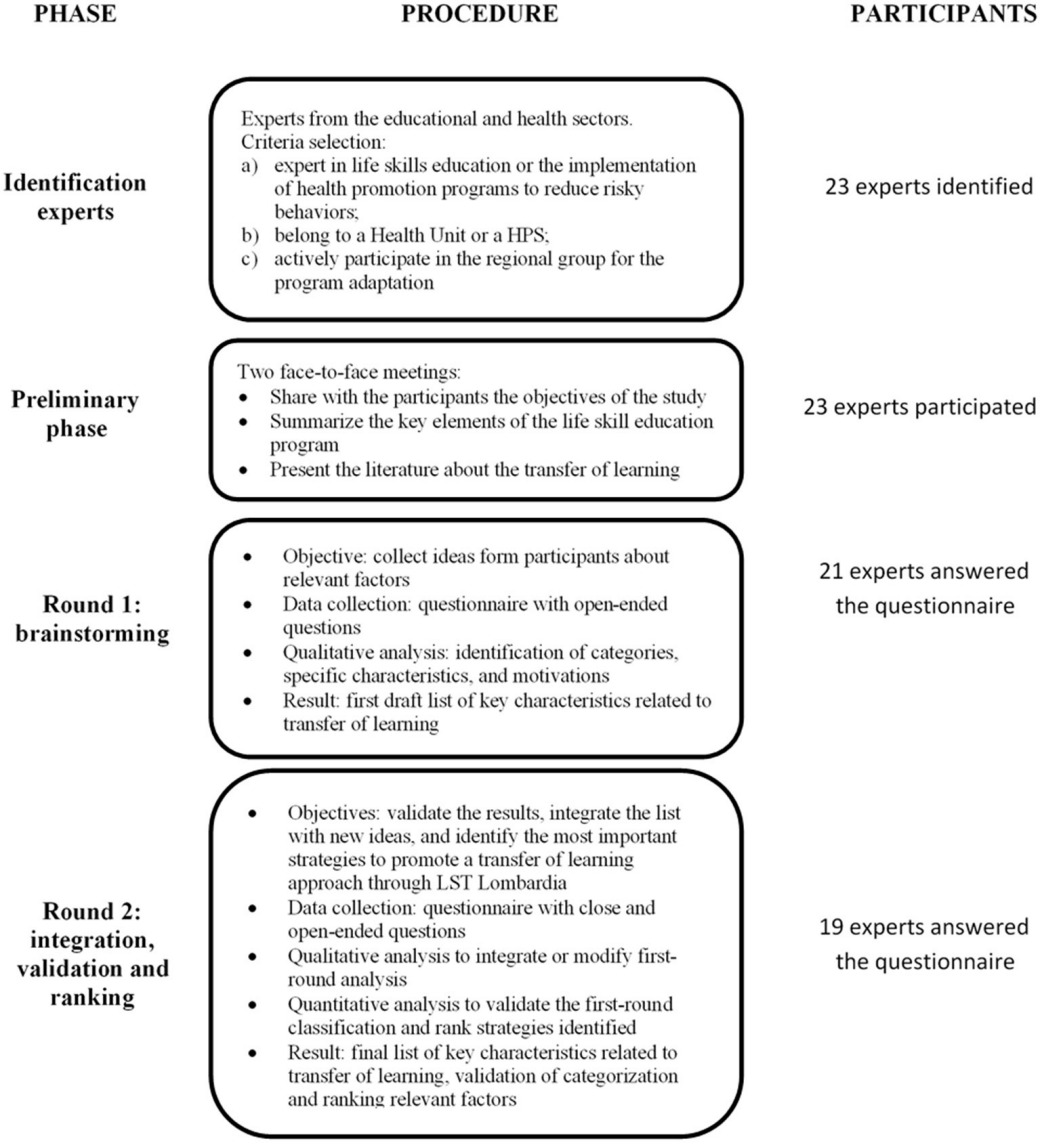
Delphi study process.

A two-round Delphi process was used in the present study as outlined by Schmidt (60, 65). First, potential experts from both the health and education sectors were identified and recruited to participate in the study. They were selected by health unit directors and HPS leaders of the Lombardy Region in the north of Italy. Inclusion criteria were that participants: (a) were experts in life skills education or the implementation of health promotion programs to reduce risky behaviors; (b) belonged to a Health Unit or a HPS; (c) actively participated in the regional group for the program adaptation. Then, a preliminary face-to-face study phase was designed to share with participants the objectives of the research and the topics being analyzed. During two face-to-face meetings, key elements of the life skill education program were summarized and an overview of the scientific literature on the transfer of learning was presented and discussed. The first-round of the Delphi procedure consisted of a brainstorming of ideas through open-ended questions. Each participant had the opportunity to write down ideas and thoughts without restrictions or limits. The authors then analyzed the answers obtained, and identified specific categories, characteristics, and motivations in participants' responses. The result of the first-round was a detailed draft list of key characteristics related to transfer of learning. A second round of the Delphi procedure was designed to expand upon and enrich the list, validate it, and choose the most important strategies for transfer of learning. The first-round results were presented to the experts involved and feedback and integration were requested. This second round also aimed to create group consensus on perspectives regarding key factors and strategies related to the transfer of learning. A third round was not necessary because data saturation was fully reached after the second round. All participants agreed with the first classification, and no new categories were added. The comments produced in the second-round provided further specification and refinement of content that emerged in the first-round. Figure 1 outlines the process of the study.

Both rounds used on-line questionnaires sent by e-mail. The study, procedure, and instruments were approved by the Regional Committee of Health Promoting School Network, and by the Regional Coordinators of the LifeSkills Training adaptation group, institutional review boards who reviewed the study for ethical standards.

### Participants

Twenty-three potential experts were identified and recruited to participate in the study. Among them, 21 answered the first-round questionnaire (91% response rate): 14 were health professionals with different backgrounds (physicians, psychologists, health workers, and educators) and with expertise in different health behaviors (addiction, bullying, mental health, nutrition, physical activity, sexual health, and sexually transmitted diseases); seven were school staff (two principals, three teachers, and two office professionals). In the second-round, 19 of these professionals answered the questionnaire: 12 from the health sector and seven from the education sector. All approached individuals agreed to participate and provided written consent.

### The Life Skill Education Program

The LifeSkills Training program (LST) (66) is a research-validated school-based prevention program proven to reduce alcohol, tobacco, drug abuse, and violence by targeting the major social and psychological factors that promote the initiation of substance use and other risky behaviors [for a review (67)]. The program provides adolescents with the confidence and skills necessary to handle challenging situations and succeed at the developmental tasks of adolescence. In particular, personal self-management skills (self-improvement, decision-making, problem-solving, coping with stress and anxiety, and managing anger), social skills (communication skills, resolving conflicts), and drug resistance skills (assertiveness, coping with peer pressure) are taught to students who receive the program. Moreover, anti-drug knowledge, attitudes against drug use, social norms awareness, and healthy values are promoted. Teaching strategies used in life skills education include traditional didactic teaching methods, facilitation and group discussion, and classroom demonstrations. A key teaching method is cognitive–behavioral skills training, which consists of instruction, demonstration, behavioral rehearsal (i.e., practice), feedback, social reinforcement (e.g., praise), and extended practice in the form of behavioral homework assignments.

In Lombardy, a region in the north of Italy, the LST Lombardia program was adapted to local culture and needs, and it was integrated with a whole school approach and HPS strategy (68, 69). Its efficacy was verified through a large-scale effectiveness study (70). It has been implemented with ~50,000 students in the region. The LST program represents an excellent case study to analyze transfer of learning because the program is recognized as a Model or Exemplary program by an array of government agencies, it is one of the most disseminated and well-known life skill education programs, and it has already been shown to be effective in changing behaviors that were not explicitly targeted (71, 72).

### Questionnaires

During the first-round, participants were asked to: (a) review summary documents about core characteristics of the LST program and an accompanying literature review about conditions and strategies to promote the transfer of learning and (b) answer three open-ended questions: (1) “Which conditions and characteristics that promote the transfer of learning are included in the LST Lombardia program?;” (2) “Which conditions and characteristics that promote the transfer of learning are lacking in LST Lombardia and should be integrated?;” (3) “Which core elements of LST Lombardia can be leveraged to facilitate these integrations?” Participants were asked to both identify conditions and characteristics and to justify their answers. Participants' names and institutional affiliations were also collected.

The second-round asked participants to read the analysis of the first-round carefully and to answer a questionnaire designed to validate the results, integrate the list with new ideas, and identify the most important strategies to promote a transfer of learning approach through LST Lombardia. Validation was assessed with three questions, with response options on a 5-point Likert scale anchored by 1 (not at all) and 5 (a lot), examining the adequacy of the coding for the first-round answers, clarity of categories, and clarity of explanations of each category and subcategory. Additional suggestions were solicited with an open-ended question to improve and refine classification. Then, participants were asked to suggest additional ideas that they might not have considered initially related to the three themes of the first-round (characteristics included, characteristics lacking, and potential elements to leverage) and to identify elements suggested by other participants that they don't agree with. These four questions were open-ended. Based on the strategies identified in the first-round, participants were asked to select the most important strategies for adapting the LST program with a transfer-oriented approach, with item response options on a 10-point Likert scale anchored by 1 (Not important at all) and 10 (Extremely important). The aim of the ranking procedure was to identify and prioritize important shared strategies for transfer-of-learning. Finally, participants were asked to make additional suggestions for achieving the tasks above.

### Data Analysis

The Delphi method establishes an iterative, multi-round process to collect systematically, aggregate, and present the individual and group's opinions or judgments on specific questions and issues related to the topic area of interest (60, 65). A two-round Delphi process was used in the present study, and results were analyzed with an integrative approach.

First-round answers were analyzed by the first author and revised and discussed with the second one. A qualitative content analysis was run (73). Answers were coded in meaningful units and then grouped under higher-order headings. Codes, subcategories, categories, and themes were identified. Both top-down and bottom-up analytic processes were used. The themes were coincident with the aims of the study. The categories, subcategories, and codes were defined by an abstraction process of answers and meaningful units. First, the units were condensed into brief descriptions close to the text of the manifest content representing the codes. The interpretation of the latent content and the abstraction process was used to define the subcategories and categories. Categories represent the program characteristics, or the strategy related to the transfer of learning. Subcategories described in-depth characteristics of specific elements related to the transfer of learning and why these elements are important.

Second-round answers were analyzed with both quantitative and qualitative methods. Qualitative content analysis was used for open-ended questions, and results were used to integrate or modify first-round analysis. Also in this case, answers were analyzed by the first author and revised and discussed with the second one. First, the answers were coded as in the first-round. Then, the new codes were compared to the first-round ones: duplicates were removed, and new codes were added and new subcategories created when necessary. No new categories emerged in the second-round, demonstrating excellent data saturation. This integrative analysis approach between the two rounds allowed the research team to aggregate the information collected during the entire study and relate responses to both individual and group perspectives. For the same reasons, the answers of experts from the health and educational sectors were analyzed together and the combination of the two perspectives were examined as a way to document consensus. This integrative approach produced a combined list of relevant factors to promote the transfer of learning. Descriptive analyses were run to validate the first-round classification and to rank strategies identified.

## Results

### Categories

Open-ended questions of the first-round were analyzed to classify the factors and strategies that facilitate the transfer of learning to different health behaviors not targeted by the LST program. The results were classified into three themes according to the study aims and 20 categories, 86 subcategories and 174 codes were identified. This classification of key characteristics related to transfer of learning was presented to participants in the second-round of the study. The experts had the opportunity to expand upon the list or to point out elements suggested by other participants that they did not agree with. No categories were added, showing excellent data saturation. The list was enriched with nine new subcategories and 17 new codes. No factors were considered as not relevant or not agreeable. Some integrations were suggested to better specify a few factors. The final list describes the conditions or characteristics of the program that can facilitate the transfer of learning to different health behaviors, the conditions or characteristics of the program that should be reinforced to facilitate the transfer, and the strategies to facilitate the transfer and adapt the program. Table 1 summarizes the number of themes, categories, subcategories and codes for each theme.

**Table 1:** Codes and categories.

	**Codes**	**Subcategories**	**Categories**
First-round: total	174	86	20
Second-round total	191	95	20
**Themes**			
Conditions or characteristics of the program that can facilitate the transfer of learning to different health behavior	78	21	4
Conditions or characteristics of the program that should be reinforced to facilitate the transfer	48	25	6
Strategies to facilitate the transfer and adapt the program	65	49	10

As shown in Table 2, four main categories of themes were identified in the two rounds regarding the conditions or characteristics of the LST program that can facilitate transfer of learning. The first category had to do with the instructional or learning methods used in the program, and several subcategories were identified that were seen as facilitating the transfer of learning. For example, the program's focus on active learning, the use of peer-to-peer practice, and the integration between practice and reflection were all viewed as key instructional methods that promote transfer of learning. The second category was related to teacher competencies that facilitated the transfer of learning. For example, the use of facilitated discussions, coaching, behavioral rehearsal, and providing positive feedback, and the use of these teaching methods across the entire curriculum were all viewed as key teacher competencies that promote transfer of learning. Also, the inclusion of questions at the end of each teaching unit to enhance students' awareness of learning outcomes and processes were considered particularly important. A third category was related to the program content and activities. Examples that facilitated the transfer of learning included the focus on life skills, the use of behavioral techniques taught to students, and the consistency with the broader school curriculum. The final category of themes identified included program characteristics that facilitated transfer of learning, and included subcategories related to the inclusion of booster sessions and the whole school approach.

**Table 2:** Conditions or characteristics of the LST program that can facilitate the transfer of learning to different health behaviors.

**Categories**	**Subcategories**	**Examples of codes about motivations**
Instructional or learning methods	• Integration of knowledge, feelings, behavior, and values in learning processes • Active learning and use of practice for learning • Peer-to-peer practice • Metacognition development • Integration between practice and reflection	• Promote the use of the body in learning • Integrate “low and high roads” • Promote student awareness of learning experiences
Teacher competencies	• Clarify objectives • Using four teaching skills during program implementation: facilitate discussions, coach, behavioral rehearsal, give positive feedback • Using questions at the end of each unit to enhance students awareness on learning outcomes and process • Students' awareness enhancement and focus on learning processes • Using of teaching skills across the curriculum • Promotion of decontextualization and recontextualization of learning	• Enhance metacognition and promote searching for connections • Offer varied practice opportunities • Promote self-monitoring • The same competencies are useful for teaching all subjects
Program contents and activities	• Life skills • Integration of knowledge, skill and attitudes/values • Practice in several domains • Meaningfulness of lessons for students • Behavioral techniques taught to students • Link with broader school curriculum	• Use a cross-domain approach • Some skills are particularly relevant for transfer of learning • Development needs are recognized • Promote the value of health • Promote self-efficacy and self-awareness • Peers offer positive reinforcement and emotional validation • Reinforce motivation • Offer behavioral schemes useful in different domains • Reinforce the same competencies needed for learning
Program characteristics	• Organization, details, and rituality • Booster sessions • Students' respect and autonomy • Whole school approach	• Offer an organization similar to curriculum lessons • Offer healthy schools routines • New practices of the same techniques promote self-awareness and thinking about skills • Booster sessions reinforce new behaviors and promote new habits formation • Promote a healthy climax and educative consistency • Value life skill culture

As shown in Table 3, six categories of themes were identified in the two rounds regarding the conditions or characteristics of the program that should be reinforced to facilitate the transfer of learning. The first category was valuing transferability, such that the program is valued as a tool for the transfer of skills. For example, the broad-based nature of the program inherently values teaching general skills and promoting youth development, not simply preventing risk behaviors. Specific elements of the program (e.g., mindfulness in learning) as well as specific abilities or skills taught in the program (e.g., self-efficacy, flexibility) were seen as critical to reinforce to effectively promote the transfer of learning. The life skill approach was also valued and identified as a precondition to transfer learning to different health domains. Other categories that should be reinforced to facilitate transfer of learning included coherence within the broader school curriculum and the focus on student needs. Participants also stated that the degree of consistency between the health promotion program and the school's planning and integration of the program within the school curriculum was critical. Finally, participants considered it important that schools explicitly promote a transfer of learning approach and establish it as an essential goal for the school. Contents about specific behavior were cited. However, participants noted that any new content should not require a new intervention, rather it should be integrated into the regular school curriculum or introduced to students through recontextualization of skills and techniques already taught.

**Table 3:** Conditions or characteristics of the LST program that should be reinforced to facilitate the transfer.

**Categories**	**Subcategories**	**Examples of codes about motivations**
Valorization of transferability	• Sensitivity and understanding of transferability • Valorization of the program as a tool to reinforce individual and social skills (not only to prevent risk behaviors) • Clarification of the conceptual framework of the program • Teacher sensitivity and competencies regarding transferability • Valorization of latent pedagogy of school routines	• Transferability is not easily understood and applied • The program can be a tool for the general development of students • Transferability depends on teacher sensitivity and educational strategies
Specific elements which promote the transfer of learning	• Decontextualization • Recontextualization • Metaphors and analogies • Mindfulness in learning • Students responsibility	• Linking learnings • Explicit wealth of meanings of learning experiences • Value personal meanings
Reinforcement of specific abilities	• Transfer considered as a competence • Self-efficacy • Creative thinking and flexibility • Digital skills	• Technologies are a new context of communication, learning, etc. • Schools need digital education
Coherence with curriculum	• Educational coherence between teacher practices and the program • Teacher teamwork • Language coherence between the program and the curriculum • Reinforcement of skill in curriculum • Transfer valorization in all school activities • Link with different subjects	• Create connections between learning experiences
Focus on students' needs	• Focus on students need • Strategies for students with special needs • Experimentation of skills in natural context and situations	• Knowledge and skills should be important for students • Activities should focus on student life realities
Attention to the context of transfer	• Focus on different physical, temporal and social contexts to which transfer can occur • Balance between the importance of maintaining a specific contextualization to learn skills and offer practices for learning in different contexts	• Skills development requires application in a specific context and reinforcement in the same context. The transfer requires contextualization in new contexts. A balance should be found.

As shown in Table 4, several categories of themes were identified regarding the conditions to facilitate transfer of learning. These included the importance of integrating LST with daily teaching, school planning, and the larger school context. This might include integrating program terminology, methods, and competencies across the entire curriculum. Other strategies to facilitate transfer of learning included explicitly valuing transferability in the program units (e.g., focusing on transferability as a competency to develop). An additional important strategy to facilitate the transfer of learning was to adapt the provider training to emphasize the potentialities of transferability and explicitly incorporate training strategies to reinforce cross-competencies. Other strategies to facilitate the transfer of learning included proposing a focus on other domains such as practice (e.g., by obtaining input and suggestions from students regarding skills practice scenarios) and new content, such as introducing specific information about new health behaviors and to focus on specific attitudes and beliefs.

**Table 4:** Strategies to facilitate the transfer of learning to different health behaviors and a ranking of their importance.

**Categories**	**Subcategories**	**Ranking—mean (sd)**
Integrate LST with daily teaching, school planning, and school context	• Include the program in the school curriculum and integrate it with different subjects • Link program objectives with EU key competences for lifelong learning • Correspondence between program terminology and school terminology • Use program methodologies also during daily teaching • Ensure that students practice life skills and program techniques during lessons • Consider the transfer of learning as a teaching style and mode of operation of the entire school • Plan school routines with a health promotion perspective and value their latent pedagogic role • Value the link between the program and school organization and routine	9.74 (0.45)
Explicitly value transferability in units	• Explicit broad objectives (not only focus on substance use prevention) • Focus on transferability as a competency to develop • Dedicate a final part in each unit to discuss how competencies and techniques learned can be useful in different domains • Refer to language and situations meaningful for students • Integrate the program with other activities aimed to apply competencies and techniques learned in other domains	9.42 (0.77)
Adapt training	• Present the LST program as a strategy to reinforce cross-competences • Present and discuss correlations between health behaviors and their determinants • Explicitly incorporate elements that facilitate transfer of learning into training • Suggest ways of transfer in each activity • Explain potentialities of transferability	9.00 (1.33)
Reinforce and integrate the questions to enhance students awareness on learning outcomes and process	• Use them in all units to promote self-awareness and mindfulness of learning experiences • Use questions to promote metacognition, abstraction, and generalization of learning • Introduce questions about the transfer of learning • Use questions to link program contents with curriculum	8.95 (0.85)
Propose a focus on other domains: practice	• Value the effects of positive practices to student self-efficacy, emotional validation, and positive peer modeling/imitation • Ensure that most examples and situations used in skills training activities refer to everyday students situations and schools relationships • Obtain input and suggestions from students regarding skills practice scenarios • Expand upon examples and situations from the program that may be applicable to multiple health domains	8.89 (1.33)
Reinforce skills	• Explicitly describe how skills are useful in different domains • Reinforce self-reflection, self-awareness, self-control and critical thinking skills • Reinforce the self-improvement project proposed in the program • Promote practice to transform knowledge and skill into competencies	8.68 (1.95)
Propose a focus on other domains: new content	• Use more metaphor and analogies to link new lessons learned to previous ones • Focus student attention to the wealth of meanings of learning experiences during units • Promote student responsibility and care • Integrate other activities aimed to introduce specific information about new health behaviors and to focus on specific attitudes and beliefs • Integrate other strategies to make context characteristics healthier and offer health behaviors practice	8.58 (1.26)
Update based on the use of technology	• Link digital skills and life skills • Include contents related to digital technologies • Consider technology and digital domains as contexts where skills are used	8.53 (1.65)
Enhance the four teaching skills	• Facilitate discussions to introduce concepts related to the transfer of learning • Coach students considering the importance of transfer of learning • Promote real situations during behavioral rehearsal • Give continuously positive feedbacks	8.21 (1.87)
Organize LST units within the context of existing school schedules	• Organize program implementation consistently with school scheduling • Define the maximal timing during the school year to implement the program to reinforce school context	8.05 (2.30)

### Validation of Categorization

All participants agreed with the list during the second-round: they reported that their own responses were well-categorized (100% rather or very well; mean 4.54; sd 0.51); that the categories were considered clear (100% rather or very well; mean 4.47; sd 0.51); and that the elements included were well-justified (95% rather or very well; mean 4.47; sd 0.61).

### Ranking Relevant Factors

The strategies identified above were evaluated by participants, and then ranked by importance. The results are reported in Table 4. All strategies were considered important, with a minimum mean of 8.05 and a maximum of 9.74. However, the ranking confirmed what was already found during the classification task.

## Discussion

In school health promotion, interventions and programs implemented often focus on single behavior domains. Typically, each health topic is addressed separately, potentially resulting in overlapping interventions. Furthermore, new health behavior problems often arise among students from year to year, requiring a response from school administrators. Rather than implementing multiple programs, it is likely to be more efficient and effective to target multiple behaviors with the same intervention or program. An integrative approach is a promising way to develop MHBC interventions, and in the present study we examined the transfer of learning approach as a strategy for expanding the scope of broad-based life skills education programs. The transfer of learning approach represents a core concept in education and is a major objective for educators and schools (44). We argue that the transfer of learning approach should be prioritized for school health promotion so that students can learn knowledge, attitudes, and skills in one health promotion context or behavioral domain and then learn how to apply them in other contexts or domains (12, 44, 45). The present study aimed to identify the characteristics of an evidence-based life skill education program that can facilitate the transfer of learning to different health behaviors not targeted by the program, the elements that should be reinforced to facilitate the transfer, and the strategies to do so successfully. A Delphi method was used involving experts in school health promotion, life skill education, and teaching. Results revealed strong consensus among experts about the potentialities of the LifeSkills Training program in promoting the transfer of skills from one health domain to another. Many elements already included in the program were identified as facilitators of transfer, and strategies to improve effective transfer were defined and clarified. These results confirm the strong potential of the transfer of learning approach in the health promotion area. Some studies have already been published (12, 44, 45), that verify the effectiveness of the transfer of learning from one health behavior to others. Life skill education has already been proven to be effective to prevent multiple health risk behaviors and promote healthy habits (47, 48), and many MHBC programs have used this strategy (8, 21, 22). However, the conditions required to effectively achieve transfer of learning have not been adequately defined and conceptualized in previous research. The findings from the present study demonstrate for the first time a consensus among experts in the field that life skills education is an adequate and promising approach for MHBC.

Most of the strategies that emerged in this study are related to teaching methods (e.g., reinforce and integrate open questions to conclude the unit or enhance teaching skills), educational approaches (e.g., explicitly valuing transferability in units), and educational consistency (e.g., integrate the life skill education program with daily teaching, school planning and school context). Contents about specific behaviors were cited, but participants suggested that they be integrated into the curriculum or addressed through recontextualization of skills and techniques already taught. Findings suggest that the successful transfer of learning to a new health domain requires that educators recognize its importance and explicitly designate it as an educational aim. These results confirmed that the transfer of learning process should consider not only the content of the program and the behavioral determinants targeted, as suggested by many authors (34–36), but also the implementation conditions. This idea is consistent with the implementation science approach which recognizes that health promotion strategies consist of complex interventions influenced by multifaceted contexts and dynamic conditions. So, the effectiveness of these strategies depends on how they are implemented, the contexts in which they are used, and the targets they reach (74, 75).

Previous studies about the transfer of learning conditions focused mainly on teaching methods and conditions (12, 34, 42, 54, 55). Their relevance was also recognized by the experts involved in this study. For example, the importance of contextualization, decontextualization, and recontextualization was confirmed (12, 34). The relevance of two paths to facilitate transfer was also recognized: the “low road” based on repeated practice and the “high road” promoted by mindful abstraction, metacognition, and deliberate search of connections (42). The study results also increase our knowledge about these teaching methods and conditions, and provide a more in-depth perspective on how teachers can implement them. For example, the “instructional or learning methods” and the “teachers competencies” already described in the LST program represent concrete strategies to promote both low road and high road paths to strengthen the transfer of learning. The “reinforce and integrate the questions to enhance students awareness on learning outcomes and process” strategy is also a practical teaching method to integrate the two paths.

Other factors and strategies identified, such as those related to educational consistency, underlined the importance of the contextual and organizational aspects to promote the transfer of learning. For example, the “coherence with curriculum” or the importance of “integrate LST with daily teaching, school planning, and school context” or of “organize LST units within the context of existing school schedules” were all related to the school organization and the curriculum definition. The categories and subcategories related to the educational approaches showed also the need to define a common and explicit educational perspective that values transferability. Again, the organizational and school community elements were highlighted by experts, which confirms the relevance of a whole-school approach to promote both educational and health outcomes promoted by the Health Promoting School approach (26–29).

There are several strengths in the present study. The use of a qualitative bottom-up approach and an integrative analysis based on the Delphi methodology helped to identify several codes and subcategories and define a rich and in-depth description of factors and strategies to promote the transfer of learning. Moreover, the involvement of experts in the health promotion area from both health educational and health sectors further enhances the elements identified and the consensus achieved among diverse experts enhances the validity of the findings. The present study has some limitations that could be addressed in future studies. A three rounds Delphi study would be more appropriate for better distinguishing the categorization phase from the validation and ranking phase. Moreover, validation could be requested for all subcategories identified. However, a preliminary face-to-face phase prepared and briefed all participants for the study, which served to increased first-round quality and facilitated participants' interactions in the second phase. Furthermore, the second-round of the study allowed all participants to comment and improve others' responses, and validation rates were high. Data saturation was reached in the second-round, and no categories were added, indicating high consensus. Another limitation was that the experts involved all belonged to the same intervention context. However, a broad range of participants from different disciplines, sectors (education and health promotion), organizations, and cultural background were selected.

## Conclusions

The present study aimed to identify the conditions necessary to effectively promote transfer of learning in an evidence-based life skill education program to different health behaviors. The use of the Delphi method findings produced a consensus among experts in the health promotion area from both the educational and health sectors. The qualitative analysis demonstrated several key necessary conditions for the transfer of learning in a health promotion context and to define them in depth.

The study confirmed the importance of investigating the transfer of learning in the health promotion area. Future studies should focus on different life skill education programs to generalize results better. Moreover, new studies are needed to evaluate the concrete effectiveness of multi behavior life skill education programs and to verify the concrete transfer of learning from one domain to another. Other Delphi studies could analyze the differences between experts from the educational and health sectors. In this study, the authors decided to explore an integrative approach to reach consensus among different expert perspectives, but an investigation of the differences can also be useful to define effective collaborative strategies.

The results have also practical implications. The identification of elements to facilitate the transfer of learning offers a solution to find a balance between the importance of applying skills to a particular topic to be effective and the need to reinforce young people in different areas of their development (48). The factors identified in this study can be used to adapt several life skill education programs to MHBC or to design new ones. The theories that underlie the LST program and the life skills targeted by it are also observed in many similar programs. Life skills programs, although more holistic in nature, also focus on knowledge, attitudes, and skills for each task or goal and these general principles are required for certain behavioral skills such as problem-solving. Moreover, the conditions identified are related to general teaching methods, curriculum definition, and school organization and could be adapted to the different programs. For example, most life skill education programs reinforce skills through specific tasks or analysis. A transfer of learning approach requires identifying general principles and rules, decontextualizing learning, and improving metacognition skills and mindful abstraction. The results of the study identified specific strategies to integrate these perspectives and practices.

The use of a Delphi study also suggests strategies to adapt programs considering experts, stakeholders and community points of view and to integrate literature guidelines with practice (76–78). Considering that most elements identified were related to educational methods and strategies, the present results can also be used to improve teacher training by promoting actions aimed to promote the transfer of learning and raise awareness of its importance. These results also provide guidance on evidence-based program implementation and integration with a whole school approach. First, the analysis of participants responses illustrates how the perspectives of health and educational experts can be effectively integrated. School staff had the chance to explicitly state their educational perspective, and health professionals were able to integrate health promotion concepts with teaching and pedagogy. Then, results show the importance of the integration of an evidence-based program with organizational and contextual elements and with program providers' representations and beliefs (68, 79–81). To integrate the program into the curriculum and make explicit the educational strategies were found to be the most valued strategies. These factors should be considered when defining the role of health professionals in supporting a school in health promotion program implementation or the HPS approach.

## Data Availability Statement

The raw data supporting the conclusions of this article will be made available by the authors, without undue reservation.

## Ethics Statement

The studies involving human participants were reviewed and approved by the Regional Committee of Health Promoting School Network, by the Regional Coordinators of the LifeSkills Training adaptation group, institutional review boards who reviewed the study for ethical standards. The patients/participants provided their written informed consent to participate in this study.

## Author Contributions

VV managed all the phases of the study. CC revised and discussed the questionnaire design and analysis. KG offered methodological support. Estensione LST group represents the coordinators of the study and the wider project. All authors contributed to the paper and involved in the study design.

## Estensione LST Group

Estensione LST group is made up of the regional staff of the project and the representatives of each organization involved: Regional Staff: Corrado Celata, Veronica Velasco, Francesca Mercuri, Sandro Brasca, Maria Grazia Crispiatico; School: Tommaso Andreano, Patrizia Bestetti, Simona Boffelli, Mara Caenazzo, Maria Concetta De Salvo, Elisabetta Franchini, Luigi Galbiati, Marina Ghislanzoni, Giancarlo Gobbi Frattini, Viviana Malvicini, Nazarena Marinoni, Amelia Molteni, Nadia Mortoni, Margherita Parolini, Umberto Parolini, Elena Pera, Cristina Pirovano, Alessandra Roncoroni, Ileana Sala Tenna, Simona Sala Tenna, Cosimo Scaglione, Alessandra Schiatti, Maria Teresa Tiana; Health Units: Stefania Bellesi, Luca Biffi, Antonella Calaciura, Rossana Di Silvio, Valter Drusetta, Laura Ferretti, Giovanni Fioni, Lidia Frattallone, Elvira Gaia, Paola Ghidini, Nicola Iannaccone, Lisa Impagliazzo, Barbara Lamera, Alessandra Maffioletti, Silvia Maggi, M. Letizia Marchetti, Margherita Marella, Raffaele Pacchetti, Ornella Perego, Giuliana Rocca, Valentina Salinetti, Cinzia Simonetti, Uber Sossi, Stefania Vizzardi, and Paolo Zampiceni.

## Conflict of Interest

KG is a consultant to National Health Promotion Associates, Inc., which markets materials for the LifeSkills Training prevention program. The remaining authors declare that the research was conducted in the absence of any commercial or financial relationships that could be construed as a potential conflict of interest.

## Publisher's Note

All claims expressed in this article are solely those of the authors and do not necessarily represent those of their affiliated organizations, or those of the publisher, the editors and the reviewers. Any product that may be evaluated in this article, or claim that may be made by its manufacturer, is not guaranteed or endorsed by the publisher.
